# 

**Published:** 1897-07-17

**Authors:** 


					THE HOSPITAL. ABSOLUTELY PURE, therefore BEST. July 1897.
CADBURY'S ^ vjf CADBURY'S
OOCOA FTP' flpj COCOA
" """""" ' NO ALKALIES USED.
isaoir W?a term Luflbmamx
No. 564. Vol. XXII. Sattoday,
July 17th, 1897.
[Snbsoription^Term8,] -p^ICE TWOPENCE.

				

